# National influenza mid-season report, 2022–2023: A rapid and early epidemic onset

**DOI:** 10.14745/ccdr.v49i01a03

**Published:** 2023-01-05

**Authors:** Myriam Ben Moussa, Steven Buckrell, Abbas Rahal, Kara Schmidt, Liza Lee, Nathalie Bastien, Christina Bancej

**Affiliations:** 1 Centre for Immunization and Respiratory Infectious Diseases, Public Health Agency of Canada, Ottawa, ON; 2 National Microbiology Laboratory, Public Health Agency of Canada, Winnipeg, MB

**Keywords:** influenza, epidemic, surveillance, paediatric, influenza A(H3N2), Canada

## Abstract

Canada’s 2022–2023 national influenza epidemic was declared in epidemiological week 43 (week ending October 29, 2022), relatively early in comparison to historical seasons. This year marks the return to pre-pandemic-like influenza circulation, following the brief and delayed influenza epidemic declared in the spring of the 2021–2022 season. To date this season, 59,459 detections of influenza have been reported out of 456,536 tests; both values exceeding historical averages. This epidemic is being fundamentally driven by influenza A, with influenza A(H3N2) accounting for 94% of subtyped detections. This season to date has had a significant impact on adolescents and young children, with a high proportion of detections occurring in those aged 0–19 years (42%). Provinces and territories have reported higher than usual influenza-associated hospitalizations, intensive care unit admissions, and deaths in comparison with previous seasons; in particular, paediatric hospitalization incidence was persistently far above historical peak levels for several weeks. The return of seasonal influenza circulation highlights the importance of sustained vigilance with regard to influenza and employment of available mitigation measures, especially of annual seasonal influenza vaccination.

## Introduction

This surveillance report summarizes the first 18 weeks of the 2022–2023 influenza season in Canada based on FluWatch data reported by the Public Health Agency of Canada from August 28 to December 31, 2022 ((1)). The national influenza epidemic began in epidemiological week 43 (week ending October 29, 2022), when the percentage of influenza tests positive exceeded the seasonal threshold of 5%. Following the brief influenza epidemic in the spring of 2022, this season is the first re-emergence of pre-pandemic influenza circulation patterns in Canada ((2–4)).

## Methods

FluWatch is Canada’s influenza surveillance system which monitors the national spread of influenza and influenza-like-illness (ILI) through core surveillance indicators based on global epidemiological standards ((5)). FluWatch consists of seven key areas of surveillance: syndromic surveillance; virological surveillance; geographic spread; outbreak surveillance; severe outcome surveillance; influenza strain characterization; and vaccine monitoring. Detailed methods, including surveillance indicator definitions, data sources and statistical analyses, can be found in the 2021–2022 National Influenza Annual Report ((2)). Pre-pandemic seasonal averages are computed using data from 2017–2018 to 2019–2020 unless stated otherwise. **Table 1** summarizes seasonal indicators up until week 52, compared with recent pre-pandemic seasons.

**Table 1:** Season indicators reported up to week 52 compared to recent pre-pandemic seasons, 2017–2018 to 2019–2020

Indicator		2022–2023	2019–2020	2018–2019	2017–2018
Epidemic onset		Week 43	Week 47	Week 43	Week 45
Onset to peak		5 weeks	14 weeks	8 weeks	14 weeks
1^st^ report of localized activity		Week 35	Week 40	Week 38	Week 36
Peak percent positivity week (%)		Week 47 (23.8%)	Week 6 (29.7%)	Week 52 (28.9%)	Week 7 (32.5%)
Dominant circulating influenza type (%)		Influenza A (99%)	Influenza B (51%)	Influenza A (99%)	Influenza A (74%)
Dominant circulating influenza A subtype (%)		H3N2 (94%)	H3N2 (68%)	H1N1 (93%)	H3N2 (96%)
Proportion of detections among ages 65 years and older (%)		26	21	16	44
Proportion of detections among ages 19 years and younger (%)		42	44	41	19
Provincial and territorial severe outcomes^a^	Cumulative hospitalization rate (per 100,000)	41	7	13	19
	Hospitalizations	3,411	618	1,064	1,493
	ICU admissions	301	73	151	114
	Deaths	182	22	27	34
Paediatric severe outcomes^b^	Hospitalizations	1,505	264	414	195
	ICU admissions	183	57	71	35
	Deaths	6	0	fewer than 5	fewer than 5
Outbreaks	Total outbreaks	534	146	86	288
	Proportion of outbreaks in LTCFs (%)	54	58	43	57

Abbreviations: ICU, intensive care unit; LTCFs, long-term care facilities

^a^ Influenza-associated hospitalizations are reported by Alberta, Manitoba, New Brunswick, Newfoundland and Labrador, Northwest Territories, Nova Scotia, Prince Edward Island and Yukon. Only hospitalizations that require intensive medical care are reported by Saskatchewan

^b^ Paediatric influenza-associated severe outcomes are reported by the Immunization Monitoring Program Active (IMPACT) network. The IMPACT paediatric sentinel hospital network consists of 12 paediatric hospitals across Canada, located in Alberta, British Columbia, Quebec, Ontario, Nova Scotia, Newfoundland and Labrador, Saskatchewan and Manitoba

### Laboratory detections

**Figure 1:**
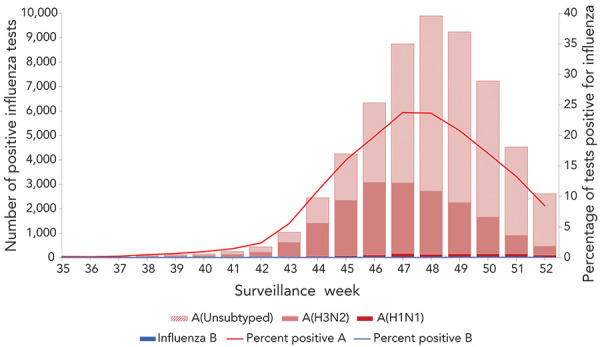
Number of positive influenza tests and percentage of tests positive, by type, subtype and report week, Canada, 2022–2023 influenza season to date, weeks 35 to 52

To date this season, a total of 59,459 influenza detections (from 456,536 tests) have been reported across the country, virtually all of which were influenza A (**Figure 1**). The number of detections at this point in the season was significantly higher than the pre-pandemic seasonal average (n=11,757) when average testing volumes were much lower (93,572 tests). Influenza A(H3N2) accounted for almost all influenza A detections (94%). Among the 37,670 detections for which detailed age information was provided, no significant differences in strain distribution were observed among age groups; however, the high proportion of detections among those younger than 19 years of age early in the season was a notable feature of this season. To date, 42% of detections occurred among those aged 0–19 years, compared with an average of 35% in previous pre-pandemic years and detections among those aged 65 years and older are within proportions previously seen.

In week 43, influenza activity surpassed the epidemic threshold, and an influenza epidemic at the national level was declared. This season’s onset occurred earlier than the historical average (week 45); however, it was not unprecedented, with the 2018–2019 national influenza epidemic also beginning in week 43. Since onset, influenza percent positivity increased sharply week to week, reaching a peak level of 23.8% (week 47), before beginning to decrease steeply in week 48. This season’s five week duration of onset to peak appears to be shorter than historical averages (12 weeks).

Among the small sample of influenza viruses submitted to the National Microbiology Laboratory (n=168) from provincial/territorial public health laboratories for antigenic characterization, all were similar to the 2022–2023 recommended Northern Hemisphere influenza vaccines, and all were sensitive to the antivirals oseltamivir and zanamivir.

### Syndromic surveillance

To date this season, all FluWatch syndromic surveillance indicators correlated with the early increases in activity reported through virologic surveillance with activity either above average levels or above expected levels typically seen in the fall and early winter. The general increase in cough and fever reported by volunteer FluWatcher participants began in week 37. To date, an average of 10,957 FluWatchers responded on a weekly basis, and the percentage of FluWatchers reporting a cough and fever remained above expected levels for five weeks (weeks 43 to 47).

The general increase in ILI activity reported by sentinel primary healthcare providers started week 42 and remained above average levels for six weeks (weeks 45 to 50); thereafter, ILI activity remained elevated but within pre-pandemic seasonal respiratory norms. Thus, ILI activity from the co-circulation of respiratory viruses including influenza, respiratory syncytial virus and severe acute respiratory syndrome coronavirus 2 (SARS-CoV-2), was comparable to the elevated levels seen during a typical pre-pandemic respiratory season ((6)). A weekly average of 46 sentinel primary care providers reported to the ILI surveillance system, seeing a weekly average of 3,276 patients.

### Severe outcomes

To date this season, 3,411 influenza-associated hospitalizations have been reported by the nine participating provinces and territories, the overwhelming majority of which have been linked to influenza A. The number of hospitalizations to date was well above the historical numbers reported at this time of year (n=1,058). Among these hospitalizations, heterogeneity exists between age groups. The highest cumulative hospitalization rates were among those aged 0–4 years (n=112/100,000 population) followed by those aged 65 years and older (n=109/100,000 population). These rates significantly exceeded both the cumulative rates among remaining age groups and the overall cumulative hospitalization rate this season (n=41/100,000 population). This season has also been marked by increased intensive care unit (ICU) admissions and deaths (301 ICU admissions and 182 influenza-associated deaths) relative to historical pre-pandemic seasons (average of 113 ICU admissions and 28 deaths), as reported by from nine participating provinces and territories. Over half (60%) of ICU admissions this season have been among persons aged 45 years and older and 75% of deaths have occurred among persons aged 65 years and older.

**Figure 2:**
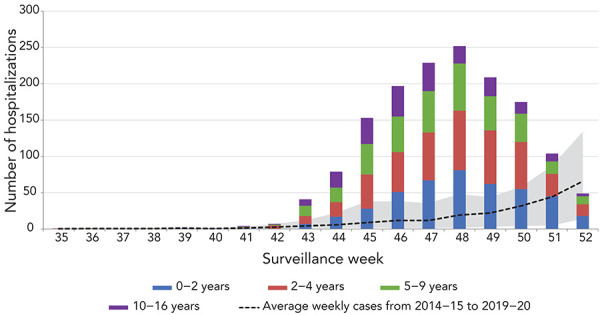
Number of paediatric^a^ hospitalizations reported by the Immunization Monitoring Program Active network, by age group, by week, Canada, 2022–2023 influenza season to date, weeks 35 to 52^b^ ^a^ 16 years of age and younger ^b^ Shaded area represents the maximum and minimum numbers of paediatric hospitalizations reported by Immunization Monitoring Program Active (IMPACT) by week from seasons 2014–2015 to 2019–2020

To date this season, paediatric influenza-associated hospitalizations reported by the Immunization Monitoring Program Active (IMPACT) were far above historic levels ever reported through the program, with 1,505 hospitalizations (**Figure 2**). The number of weekly hospitalizations began to spike in week 42 before reaching a peak of 252 in week 48. Weekly incidence counts reported from week 45 onward (range 153–252 hospitalizations) exceeded the all-time season peak previously reported (n=151 in week 9 of the 2015–2016 season) for six consecutive weeks. Cumulatively, the number of paediatric influenza-associated hospitalizations at mid-season exceeded all prior annual/full-season numbers. Virtually all hospitalized cases were due to influenza A, and among subtyped cases (n=584), 94% were associated with influenza A(H3N2). The largest proportion of hospitalized cases was among children between the ages of two and four years (32%), followed closely by children between the ages of five and nine years (24%). Paediatric ICU admissions to date were also above historical averages, with 183 reported this season compared to an average of 54 in previous seasons. Approximately 12% of hospitalizations resulted in an ICU admission this season and children between the ages of two and four years and five and nine years accounted for 31% and 22% of paediatric ICU admissions respectively. Influenza-associated paediatric deaths were also higher than previous seasons, with six influenza-associated paediatric deaths being reported thus far.

### Outbreaks

To date this season, 534 laboratory-confirmed influenza outbreaks have been reported. The number of outbreaks reported was higher than historical numbers reported at this time of year (n=173). All but one outbreaks reported were due to influenza A. Outbreaks reported in long-term care facilities accounted for the highest proportion of outbreaks (54%), followed by facilities classified as “other” (28%) ((3)). Among the 231 ILI outbreaks reported, nearly 99% occurred in schools/daycares.

## Discussion

Following the short and delayed influenza A(H3N2)-dominant 2021–2022 influenza season, Canada has seen a return of late fall influenza activity resulting in a seasonal epidemic. This season, which began early relative to historical seasons, has since demonstrated a steep progression and significant impacts on the paediatric population.

The length of the season is difficult to infer based on the timing of onset, as historical data points to differing trajectories. Factors such as the timing of the peak as well as the proportion of influenza A and B circulating have a bearing on the duration of the influenza epidemic. On average, historical pre-pandemic seasons have peaked at 30.4% between week 52 and week 7, in contrast to what has been seen so far this year. To date, the seasonal epidemic has been driven by influenza A(H3N2), with minimal circulation of influenza B. Contrary to previous pre-pandemic seasons, we have yet to see an increase in the relative proportion of influenza B detections. It is currently unknown whether Canada will see a typical late season wave of influenza B.

To date this season, trends in the severity of influenza cases have been heterogeneous between age groups. Paediatric hospitalizations reported by the IMPACT network were far above historically seen levels. Explanations behind this phenomenon are complex and difficult to untangle. The coronavirus disease 2019 (COVID-19) pandemic response disrupted seasonal respiratory virus transmission across the nation and resulted in a large unexposed cohort of young children who may be more vulnerable to severe infection. For instance, the IMPACT network reported no paediatric hospitalizations, ICU admissions or deaths throughout the 2020–2021 season ((7)). The cessation of previously mandated non-pharmaceutical interventions, such as mask wearing, may have facilitated the increase of transmission in the community ((8,9)). The lifting of travel and border measures may have allowed the re-introduction of seasonal influenza to Canada from regions where community circulation was occurring ((10)). When looking at the relative proportions of hospitalizations by age group, it is interesting to note that distributions are unusual given the predominance of influenza A(H3N2), a pattern that carries on from the late, short 2021–2022 season. Similar to the short epidemic experienced in the spring of the 2021–2022 season, to date, a higher proportion of detections and activity were among children and teenagers, who have typically experienced a lower proportion of detections and activity during influenza A(H3N2)-dominant seasons ((2)).

The beginning of seasonal vaccination campaigns coincided with the early onset of the seasonal influenza epidemic. Regardless of the timing of this season’s peak percent positivity, influenza circulation is expected to persist for many weeks. In previous seasons, the progressive decline to levels below the epidemic threshold after reaching the peak has taken an average of 20 weeks (2016–2017 to 2018–2019). It remains important to seek vaccination in the face of the ongoing epidemic. Antigenic and genetic characterization results received to date suggest that the circulating strains of influenza A(H3N2), A(H1N1) and B are similar to the recommended Northern Hemisphere vaccine components for the 2022–2023 season. The vaccine effectiveness (VE) of the 2021–2022 vaccine against the current circulating A(H3N2) sub-clade was moderate (36%); however, this season’s H3N2 component appears to be more antigenically similar to currently circulating strains ((11)). Although antigenic similarity is not a consistent predictor for vaccine effectiveness, which is dependent on several factors ((12)), preliminary findings from the Canadian Sentinel Practitioners Surveillance Network (SPSN) based on data up to week 50 indicate that the risk of medically-attended H3N2 illness was approximately halved among recipients of the current season’s vaccine compared to unvaccinated individuals ((13)).

The early and relatively intense resurgence of influenza highlights the importance of continued seasonal influenza surveillance. The systematic collection of influenza surveillance data has enabled the situational awareness to respond to the current influenza season in the context of the ongoing COVID-19 pandemic. Additionally, the use of the same indicators as those used prior to the COVID-19 pandemic has allowed for the interpretation of both the magnitude and the spread of influenza in the 2022–2023 season. Ongoing, timely surveillance is crucial to Public Health Agency of Canada’s ability to respond to influenza trends, monitor changes in circulation patterns, and facilitate preparedness for and planning of mitigation measures within the influenza season.
